# Chromatographic Fractionation of *Penicillium polonicum* Fermentation Metabolites in Search of the Nephrotoxin(s) for Rats

**DOI:** 10.3390/life12050747

**Published:** 2022-05-18

**Authors:** Ana Miljkovic, Peter Mantle

**Affiliations:** Biochemistry Department, Imperial College, London SW7 2AZ, UK; anabrake@gmail.com

**Keywords:** apoptosis, glyco-peptide, proline, electrospray mass spectrometry, ninhydrin, ochratoxin A

## Abstract

Complex renal histopathological changes in rats, in silent response to dietary contamination with wheat moulded by a common *Penicillium* from the Balkans, have long eluded attribution of a causal toxin. So far, water-soluble amphoteric glyco-peptides seem responsible, at least for the nuclear pyknoses in nephron epithelia after several days of dietary exposure. Recently, refined histology analysis has diagnosed pyknosis as apoptosis, and followed the finding through application of medium-pressure liquid chromatography, anion exchange and silica layer chromatography to fractionate a water/alcohol-soluble extract of a fungal fermentation on wheat. Proline was revealed, with other amino acids, in acid hydrolysate of the fermentation extract. Application of mass spectrometry has recognized prominent ions (*m*/*z* 550 and 564) correlated with fragmentations consistent with a terminal proline moiety for the putative toxins, coupled with other structural fragments and correlated with apoptosis. Use of ^14^C-proline in probing *Penicillium polonicum* fermentation to aid isolation of the new potential toxins, along with application of gel electrophoresis, may further aid characterization of the apoptosis toxin(s). The present focus on proline peptides in mycotoxicosis fits easily with their increasingly recognised pharmacological activity associated with proline’s rigid secondary amine structure, which causes conformational contortion in peptides. Nevertheless, there remains the striking rat renal karyocytomegaly by *P. polonicum*, for which there is yet no causative mycotoxin.

## 1. Introduction

Fungal metabolites that specifically target mammalian kidneys are unusual. The first was ochratoxin A [1], discovered and characterised in South Africa while aflatoxin was being recognised as a general carcinogen; both contributed to establishing the term mycotoxin. The moulds concerned were among species of the widespread genera *Penicillium* and *Aspergillus*. Ochratoxin A quickly found relevance to a porcine nephropathy, seasonally endemic in the Danish bacon industry in the mid-20th century [2]. Lateral thinking [3] connected it hypothetically to the curious Balkan Endemic Nephropathy (BEN), first recognised as an entity in the early 1950s. Concurrently, in spite of geo-political fractures, limited mycological study was made in the UK on fungi sourced from Balkan villages in which the silent renal atrophy was hyperendemic, even to obtain data hypothetically to link seasonal mortality from the BEN with annual rainfall patterns [4]. Focus was made on common food spoilage fungi, isolated examples of which were explored for toxicity in a collaborative study in London, resulting in a seminal publication [5]. Measurement of cellular toxicity in vitro, and marked renal histopathological changes found in rats given cultured biomass of all three examples of a *Penicillium* sp. abundant in hyperendemic villages, raised the possibility of its relevance to the aetiology of BEN. More recently, this fungus has been recognised as *P. polonicum* Zaleski [6].

In the first search for its nephrotoxin(s) [7], fractionation strategies for isolating potential toxins from this fungus, grown on yeast extract/sucrose medium in stationary liquid culture for 2 weeks, commenced with focus only on fungal biomass homogenised in water. Filtrate was evaporated, lyophilised and aliquots tested positively for toxicity in vitro and in rats in vivo. For the former, kidney cells were grown to confluence, the medium replaced with a test solution of fungal extract, incubated for 4 h, [1-methyl-^3^H] thymidine added for 1 h, cells washed and ^3^H incorporation measured against controls to assess cell viability. To assess renal histopathology, male Sprague–Dawley rats were first given feed containing cultured fungal mycelium for 2 days. Concurrently, lyophilised mycelial extract was re-constituted in water (pH 4) and treated with cation exchange resin; non-exchanged material proved non-toxic. Similarly, the cation-exchanged material was treated with anion-exchange resin and, again, toxin(s) proved to be retained on the resin. The anion-exchanged material was then fractionated via size-exclusion chromatography to demonstrate a size, ~1500 daltons. 

Aliquots (20–40 mg) of the toxic size-selected compound fraction in water were passed through reverse-phase Sep-pak cartridges (Waters Associates) and eluted with water or acetonitrile + water or acetonitrile. Toxicity was confirmed for the first two eluants, but mainly for aqueous acetonitrile, giving a complex pattern in HPLC according to UV-detection (λ—226 nm). Notably, sub-Fraction 3, consequent on a step-change in elution solvent, demonstrated toxicity in in vitro assay and was fractionated further via HPLC to four distinct components. Hydrolysis of each, and amino-acid auto-analysis, showed the three most prominent to be rich in Asp, Glu, Gly and Lys, indicating a family of peptides together representing ~0.02% of original fungal biomass. 

The size-exclusion fraction, following anion exchange above, was also processed through the chromatography of high-voltage preparative electrophoresis (HVE) on Whatman 3MM paper, loaded in a pH 6.5 environment mid-way between anode and cathode. After 40 min in toluene coolant at 3 kV, edges of the paper were sprayed with cadmium-ninhydrin reagent to reveal primary or secondary amino groups migrating towards either electrode, with one in the neutral centre close to a dansyl-arginine marker. Only the latter gave the typical rat renal histopathology after administration in feed.

In a second research programme [8,9], the search for the nephrotoxins focused on preparative HVE after culture of a freshly isolated Yugoslavian fungus attributed by the current taxonomy to *P. aurantiogriseum*. The typical expression of rat nephrotoxicity was confirmed by the histopathological changes following administration directly by adding whole culture to feed and indirectly via a crude ethanol extract. The latter was also fractionated by preparative HVE on paper, again selecting a ninhydrin-positive band and then isolating negatively charged components via anion exchange resin. Aliquots (85 mg) of the freeze-dried fraction, given daily for 4 days to a rat, via either feed or intraperitoneal injection in water, caused the renal histopathology, with changes for the latter being particularly severe. This is not surprising, considering delivery near kidney, but leaves open a question about any alimentary degradation. Similarly, failure per os of *P. polonicum* to cause renal pathology in hamsters [10] and primates [11] might simply reflect bioavailability. The only other study seeking the nephrotoxins, although set in the histopathology of the of the renal karyocytomegaly aspect of *P. polonicum,* also used the combined mixture of cation exchange, HVE and HPLC to provide putative toxic fractions for rat bioassay. A variation in HVE was the ability to sew an excised paper band from an electrophoretogram developed at pH 6.5 on to a new paper, subsequently run similarly but at pH 2.1. This effected further separations, yielding five nephrotoxins, each rich in either ser, pro, val or leu. These differed from those from HVE (pH 6.5 only) [8], but a wider extent of peptides within the product of sequential chromatographic separation of *P. polonicum* extrolites became apparent.

A recent illustrated summary [6] of rat renal histopathology in response to oral intake of *P. polonicum* metabolites, verifying apoptosis also for the mycotoxin ochratoxin A, provides open access to the background for the present report.

## 2. Experimental

### 2.1. Confirming Typical Renal Pathology for a P. polonicum Fermentation

Profusely sporulating fungal mycelium, grown on shredded wheat, was covered with 95% ethanol (250 mL/ 1 L conical flask), shaken and extracted overnight. The mixture was vacuum filtered through filter paper (Whatman No 50). The residue was re-extracted, first with more alcohol and then with water and combined filtrate evaporated to a brown viscous solution. Transferred to 4 °C, the mannitol generated metabolically during fermentation, was extracted in a crystalised state by the aqueous extraction step and the decanted supernatant was divided into 1 quarter or 3 quarters portions. Each was homogenised into 100 g powdered rat diet for consumption by a 250 g male rat over 5 days. For the lower dose, the accumulated intake represented extract of 15 g of moulded shredded wheat and the larger portion represented 45%. The histopathological effect (haematoxylin and eosin staining) on kidney showed typical changes, i.e., pyknotic nuclei and nuclei with condensed and/or fragmented chromatin resembling “mitotic figures”, confined to the cortico-medullary region and confirmed also as apoptosis by ApopTag^®^ fluorescent staining, as previously described [6]. Changes were more abundant in the rat with the higher dose, confirming at least a general dose/response relationship for kidney pathology for subsequent steps toward characterising the nephrotoxin(s).

### 2.2. Application of Medium-Pressure Liquid Chromatography (MPLC) for Fermentation Extract Fractionation

Aqueous extract, diluted with an equal volume of chromatography running solvent (25% acetonitrile in water containing 0.01% trifluoroacetic acid), was injected (3 mL) into a Buchi pre-column (B-165, 115 × 9.6 mm) by a Buchi B-688 pump at 4 mL/min prior to the main C_18_ silica reverse-phase column (230 × 26 mm). Separation was followed in eluant via a Pye Unicam UV detector (PU 4020) at λ = 214 nm to detect peptide bond absorption displayed on a Kipp and Zonen chart recorder at 2 mm/min.

**Figure 1:**
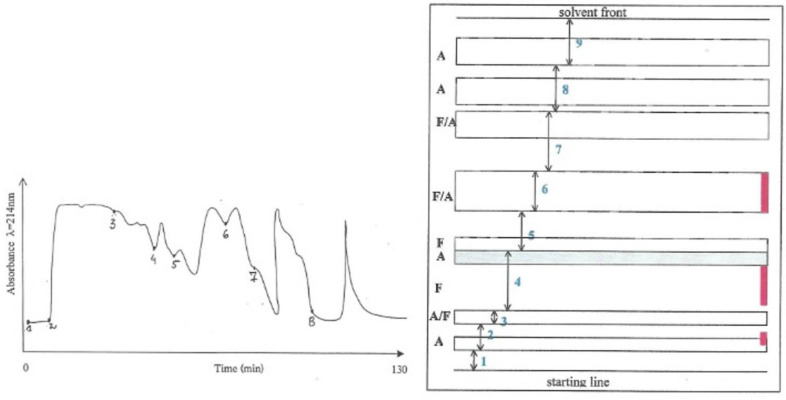
(**Left**). Absorbance pattern of the water-soluble fraction and its fragmentation into 8 sub-fractions obtained by isocratic (25% acetonitrile in water) medium pressure liquid chromatography (numbers indicate the point at which each fraction commences). (**Right**). Schematic presentation of a preparative layer chromatography plate showing the fractionation pattern of sub-Fraction 2 (A—absorbance at λ = 254 nm, F—fluorescence at λ = 350 nm). Ninhydrin-reactive regions in red.

Thus, 3 g of the water-soluble fraction, which theoretically corresponded to an equivalent of a 20% *P. polonicum*-moulded diet for 5 days for a rat, was fractionated into 8 parts according to the absorbance pattern (Figure 1, left) in an isocratic system (25% acetonitrile in water) and fractions were collected individually. According to previous studies [8], Fraction 2 could be expected to carry most of the nephrotoxicity.

Another 3 g of the water-soluble fraction were also subjected to MPLC, and all eight fractions eluted from the column were combined.

Fraction 2 alone from the first fractionation, and the combined eight fractions of the second fractionation, were bioassayed for dietary nephrotoxicity, each in an adult (200 g) male rat for 5 days. In parallel, the renal histopathological response was compared with that caused in a third rat by the whole original crude alcohol/water extract (3 g) of *P. polonicum* fermentation. All three rats showed prominent and almost equally frequent histopathology response. This indicated that the nephrotoxic compound(s) could be eluted from the reverse phase C_18_ column under the utilised conditions and that most of the nephrotoxicity was confined to Fraction 2.

### 2.3. Isolation and Purification of Penicillium polonicum Nephrotoxin(s) from the MPLC-Fraction 2 by Preparative Layer Chromatography

**Figure 2:**
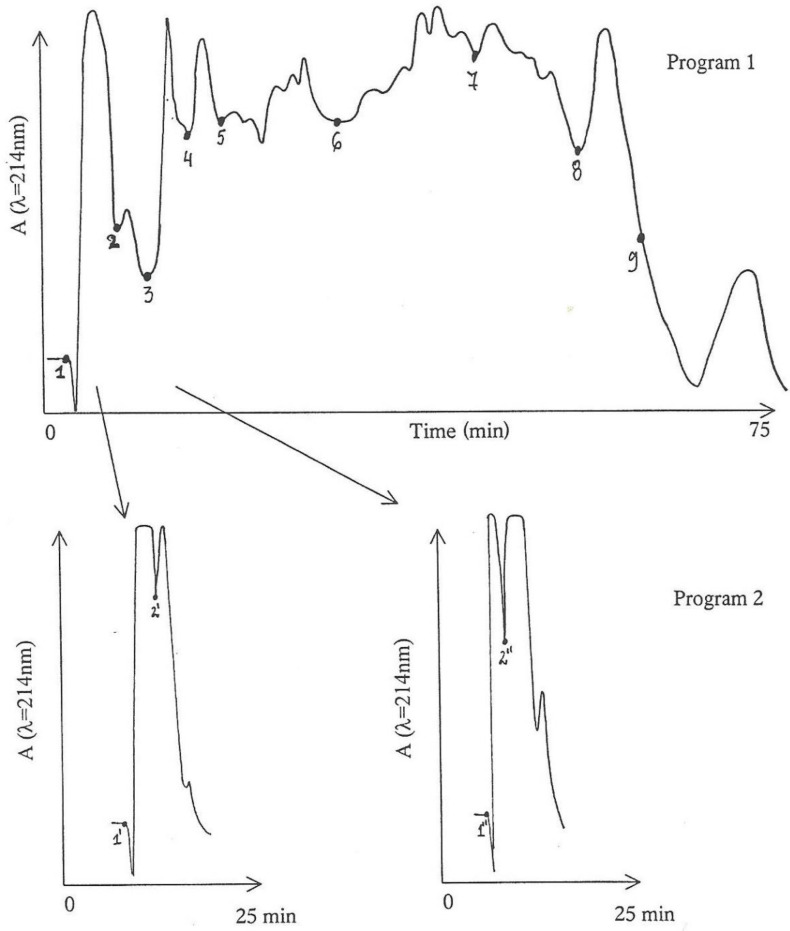
Absorbance pattern of the active MPLC-Fraction 2 and its further fractionation by gradient MPLC into 9 further fractions by Program 1 and four additional sub-fractions (by Program 2). Numbers indicate the point at which each fraction commenced.

Fraction 2 (in 2.2 above) was further fractionated by preparative layer chromatography (PLC) (Figure 2). An amount of Fraction 2, that theoretically corresponded to a 20% *P. polonicum*-moulded diet for 5 days to a rat, was applied to SIL G-200 UV _254_ PLC plates (Camlab) and developed with n-propanol:ammonium hydroxide (1.84 S.G.) (7:3 vol/vol) for 15 h. The separated components were divided into nine bands, according to the patterns of absorbance and fluorescence as seen at λ = 254 nm and λ = 350 nm, and edge-stained with ninhydrin as illustrated in Figure 1, (right). After elution of the components from excised silica regions with water/methanol/ammonia (4.5:4.5:1), Fractions 2–7 were bioassayed for nephrotoxicity. A typical prominent response was found in the kidney sections of the rat given Fraction 2, whilst only a few nuclei with condensed chromatin (“mitotic figures”) were observed in the kidney sections of the rats given Fraction 3. No response was induced by any of the other fractions. This indicated that most of the nephrotoxic activity was, again, present in Fraction 2.

### 2.4. Further Fractionation of the Active Anion-Exchange Fraction Obtained from MPLC-Fraction 2 by MPLC-Gradient System

More of the active anion-exchanged MPLC-Fraction 2, equivalent to that in 2.3 above but in threefold greater amount, was further processed by the MPLC-gradient system in water containing 0.1% trifluoroacetic acid and 5% acetonitrile for five minutes. Acetonitrile was then increased linearly to 30% during the next 45 min and maintained at 50% for the next 20 min (Figure 2, Program 1) to complete the elution. A less steep gradient was employed for Program 2, further in another batch to stretch the first 15 min of fraction 1. For Program 2, the initial acetonitrile concentration of 5% was increased linearly to 10% over 20 min and maintained at that for a further 15 min.

Components eluted from Program 1 were divided into nine fractions according to the absorbance pattern. Assuming that nephrotoxic activity was in the earlier-eluted components, Fractions 1 and 2 were further fragmented into two sub-fractions via Program 2 (Figure 2); consequently, sub-fractions 1′ and 2′, and 1″ and 2″ were obtained from the point of marking in Figure 2, respectively.

Each of the four sub-fractions (1′, 2′, 1″ and 2″) and Fractions 3–9 were bio-assayed for nephrotoxicity in small (80 g) rats, mostly from the same litter in the hope of obtaining the most even dose response. The histology was compared in a positive control, i.e., a rat treated with a 20% *P. polonicum*-moulded diet. The original non-anion-exchanged fraction was also bio-assayed. 

Additionally, each of the above sub-fractions and fractions were briefly explored on TLC in comparison with the original (non-anion-exchanged) MPLC-Fraction 2. Chromatograms were sprayed with ninhydrin reagent; the distribution of ninhydrin-reactive compounds revealed that a fine and gradual sequence of components were separated during the gradient MPLC. 

The kidney sections of the rats showed the following responses:

1′ Normal.
2′ Few condensed nuclei in eosinophilic cytoplasm at the cortico-medullary region (CMR).
1″ Normal.
2″ Fairly extensive number of condensed eosinophilic nuclei at the CMR.
3 Very extensive number of condensed eosinophilic nuclei at the CMR.
4 Fairly extensive number of condensed eosinophilic nuclei at the CMR and in the outer medulla.
5 Very occasional “mitotic figures” amongst a few condensed eosinophilic nuclei at the CMR.
6 Occasional presence of “mitotic figures” amongst a few condensed eosinophilic nuclei at the CMR.
7 Extensive number of condensed eosinophilic nuclei at the CMR.
8 Normal.
9 Normal.

Positive control—typical and prominent response

Non-anion-exchanged fraction—normal

Rats given Fractions 1′–8 and the positive control were all from the same litter.

It was concluded that nephrotoxicity was distributed across Fractions 2 (2′ and 2″)–6, which indicated that at least some nephrotoxicity had been retained to support recognition during the further fractionation of the moderately active anion-exchanged fraction. However, the intensity of the response observed concerning these fractions was weaker than that in the positive control, suggesting that some activity has been obscured during the necessary dissection of nephrotoxins.

Apoptosis was also explored by staining kidney sections with ApopTag^®^ Direct Labelling Kit [6]. A few fluorescent apoptotic bodies were observed in the cortico-medullary region in the positive control rat and in the rats given Fractions 2–4. Nevertheless, although this did not greatly contribute towards better differentiation of the nephrotoxicity amongst the fractions, it suggested that weanling rats might not be reliable for exploration of the apoptotic effect caused by *P. polonicum* nephrotoxins if age was critical.

**Figure 3:**
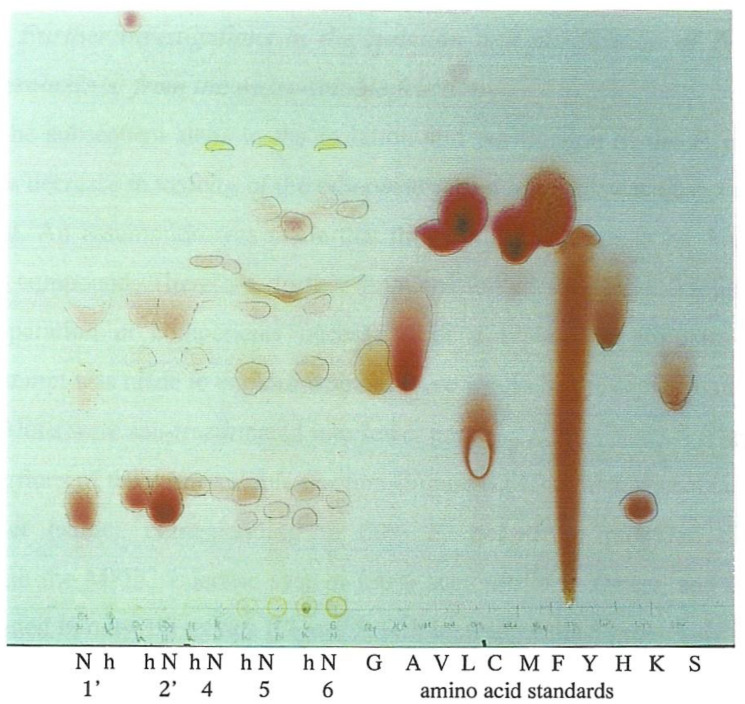
Silica gel chromatography of samples of fermentation Fractions 1′, 2′, 4, 5 and 6 (N) compared with hydrolysed aliquots (h) of the same, and with reference to some amino acid standards. Sprayed with ninhydrin reagent. Note the yellow high Rf value spot, each for hydrolysed Fractions 4–6, attributed to proline.

Furthermore, previous investigations of the chemical nature of *P. polonicum* nephrotoxin(s) indicated that it/they might be peptides or glycopeptides [8]. Thus, to explore whether any of the fractions contained amino-acid-based compounds, a small portion of each fraction tested was acid hydrolysed prior to amino acid analysis. Thus, ninhydrin-reactive components from the hydrolysed Fractions 1′, 2′, 4, 5 and 6 were compared with those from non-hydrolysed material and some amino acid-standards by TLC (Figure 3). This showed that the hydrolysis caused changes in the number and Rf values of some ninhydrin-reactive components. Thus, for example, three spots in the non-hydrolysed components become 6 spots in the hydrolysed components in both Fractions 5 and 6. Some of the ninhydrin-reactive components on the plate were matching glycine, valine, leucine, methionine, histidine or lysine, in colour or Rf value. Most notably, proline was strongly suggested by three yellow spots of high Rf value correlated with hydrolysed Fractions 4–6. In retrospect, inclusion of Fraction 3 could have been interesting.

### 2.5. Further Investigations in the Isolation and Purification of Penicillium polonicum Nephrotoxin(s) from the Water-Soluble Fraction

During the subsequent steps in the isolation and purification of the *P. polonicum* nephrotoxin(s), a decrease in toxicity as indicated in H&E-stained histopathology of the otherwise active fractions was observed at each succeeding stage. An assumption was made that the nephrotoxicity may be due to more than one active compound. Therefore, further fractionation of the active fractions might have led to separation of components necessary for a corporate nephrotoxic response. Therefore, an attempt was made to explore whether there would be the same loss of activity if the active fractions were sub-fractionated into fewer parts. 

**Figure 4:**
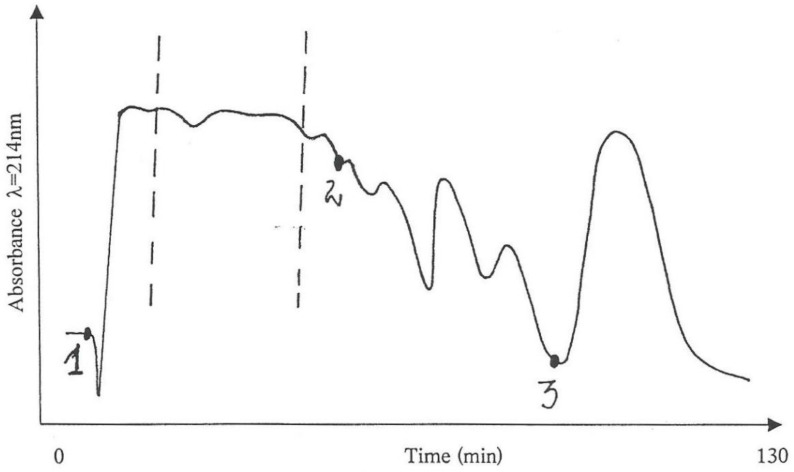
Absorbance pattern of the water-soluble fraction and its fragmentation into 3 sub-fractions obtained by isocratic (50% acetonitrile in water) MPLC. Numbers indicate the point at which each fraction commences.

Thus, portions of the water-soluble fraction that was obtained from the initial alcohol/water extract, corresponding to a 60% *P. polonicum*-moulded diet, were processed through the MPLC isocratic system (50% acetonitrile in water) and the eluted components divided into only three fractions (Figure 4), instead of the eight previously described, which were tested for nephrotoxicity in three male rats (200 g), together with a positive control rat that was given an analogous amount of the “original”, i.e., non-fractionated water-soluble, fraction.

The bioassays showed typical histopathological changes in the rat given Fraction 1, while no changes were seen in the rats given Fractions 2 and 3. However, the intensity of the positive response in the rat given Fraction 1 was less than that in the positive control rat, confirmed by quantifying average cortico-medullary histopathology across seven × 40 fields, contrasting 13.5 abnormalities for Fraction 1 with 45.5 in the positive control. Then, to seek for the lost activity, Fractions 1–3 were recombined and tested for a possibly increased nephrotoxicity in comparison with control rats given solely Fraction 1 or the non-fragmented (“original”) water-soluble fraction. However, the intensity of the response remained the same as in the rat given only Fraction 1, but still weaker than that given the whole fraction. We were, however, conscious of animal variation and both economy and ethics of excessive bioassay. Nevertheless, this apparent loss of activity, even in the case of rather conservative fractionation, indicated why the problem of isolation of the active compound(s) is so challenging.

**Figure 5:**
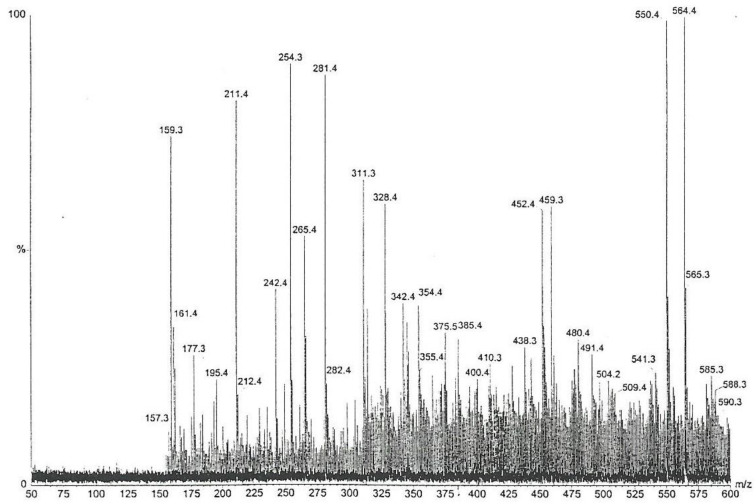
Mass spectrum (electro spray +1) of the sub-fraction of Fraction 2, showing the prominent pair of methyl analogues *m*/*z* 550 and *m*/*z* 564.

**Figure 6:**
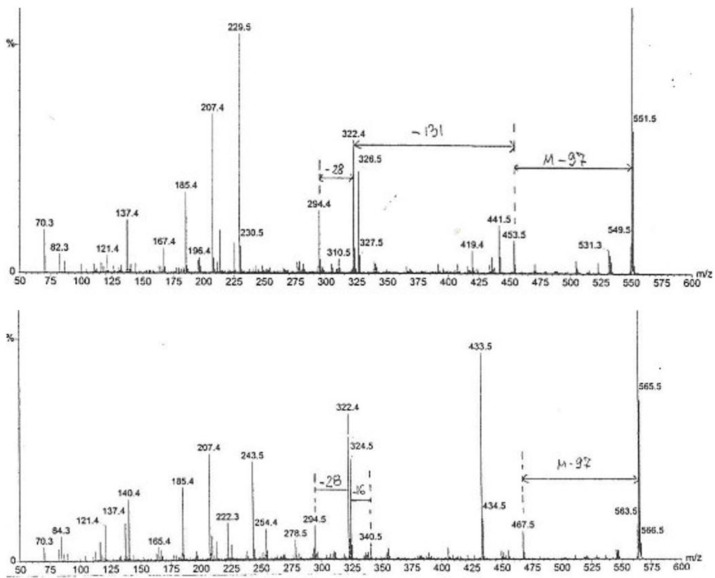
MS/MS spectra (electro spray +1) of the methyl analogues: above with molecular ion *m*/*z* **550**, showing loss of 97, 131 and 28 mass units equal to proline, methionine and 2xCH_2_, respectively; below with molecular ion *m*/*z* **564**, showing loss of 97, 16 and 28 mass units equal to proline, NH_2_ and 2 CH_2_, respectively.

Furthermore, the chemical complexity of the active Fraction 1 was explored; a sub-fraction of Fraction 1 (as labelled by vertical lines in Figure 4), which did not crystallize in cold, i.e., contained little or no mannitol, was analysed by high-sensitivity collisionally activated decomposition tandem mass spectrometry [12]. The electrospray data (Figure 5) notably show, amongst other molecular ions or fragments, a pair of methyl analogues *m*/*z* 550 and *m*/*z* 564 that had mutually similar fragmentation patterns and/or fragments (Figure 6). Some of the mass differences corresponded to the loss of amino acids, such as proline or methionine, which indicated that these compounds might be peptides. In any case, the even number molecular ions in the positive mode showed that compounds contained at least one nitrogen atom. 

### 2.6. Apoptosis Induced by the Active Fraction 1

**Figure 7:**
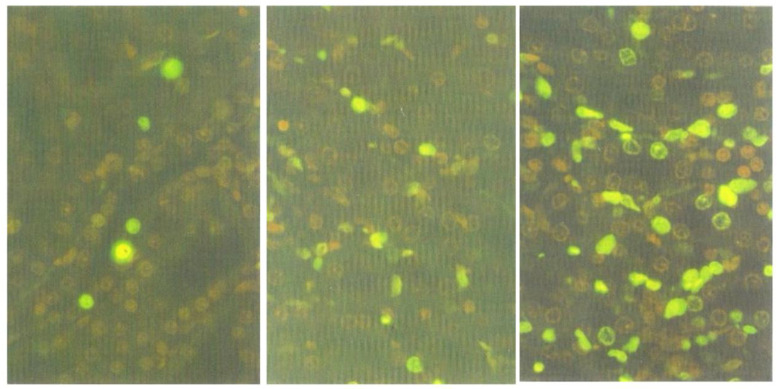
Micrographs of rat kidney sections showing fluorescent apoptotic bodies in the inner cortex (no glomerulus), (**left**); cortico-medullary region, (**centre**); outer medulla, (**right**). ApopTag stained, ×200.

The decrease of the nephrotoxic activity in Fraction 1 (section above) indicated that some components, necessary for the induction of typically prominent response, might have been lost during the fractionation procedure. Thus, to explore whether the nephrotoxic factor(s) responsible for apoptosis had remained in the Fraction 1, kidney sections of the rat tested for this fraction were stained with ApopTag^®^ Direct Labelling Kit. Many apoptotic cells were found in the outer medulla (Figure 7, right), whilst fewer were present in the cortico-medullary region (Figure 7, centre), and only a few in the inner cortex (Figure 7, left). Whereas it was previously shown [4] that, after treatment with standard *P. polonicum*-moulded diet or crude extract, most of the fluorescent apoptotic cells were confined to the cortico-medullary region and were rarely in the medulla, the target nephron epithelia for apoptosis are not all confined to a narrow ‘cortico-medullary’ region. Therefore, after several days of exposure to the nephrotoxin(s), apoptosis could be expected to have flowed further into the medulla.

## 3. Discussion

The key coordinated findings here are the consistent evidence of proline in acid hydrolysate of separated nephrotoxins and the diagnostic indication of a 97-mass-unit (prolyl) loss in the first MS fragmentation from two prominent MS ions, 550 and 556. Indication of methionine as another amino acid component further adds to the perception of peptides. Application of ^14^C radiolabelling to biosynthetic analysis [13] involving proline could be of prospective value; it would first require fermentation refinement of *P. polonicum* for a liquid medium and some timeline for optimum nephrotoxin production as shown by rat renal bioassay. This should facilitate isolation of nephrotoxin(s) for more spectrometric, particularly for accurate mass measurement, and spectroscopic data collection. Accurate mass might help verify the sulphur atom in a methionine component.

Another technique that was highly valued for earlier studies [8] is high-voltage electrophoresis on paper cooled in toluene, as a preparative facility. This was designed in the new biochemistry building at Imperial College in the 1960s but later discontinued, possibly for safety reasons. Maybe such is still available elsewhere, or available in a gel context, to further progress the present topic. This could be a strategy towards purifying enough of the fungal products for structural verification to be achieved.

The question of the extent to which glucose could be an integral part of the nephrotoxic peptides was separately addressed by demonstrating that glucose could be detected in partially fractionated fermentation extract by standard paper chromatography, and that a perfect ^13^C NMR spectrum could be obtained. Thus, opportunity for linkage with a small peptide exists in a *P. polonicum*-moulded wheat substrate.

When proline is involved in a peptide bond, it does not have a hydrogen atom on the alpha amino group and cannot donate a hydrogen bond to stabilise in a helix or beta-sheet. In a helix, there will be a slight bend due to the lack of the H bond. As of 18 years ago, only 5% of the 130 extrolite families produced within *Penicillium* subgenus *Penicillium* contained proline as a biosynthetic component [14], but none appear to be analogous with the present *P. polonicum* water-soluble peptide(s) that are likely causing renal apoptosis in rats. The recently described amino acid construct, purpurolic acid [15], uniquely combining proline and alanine moieties, is also water soluble, as shown by its original discovery [16] via ion-exchange liquid chromatography for free amino acid quantitation [17]. Proline peptides are becoming of increased pharmaceutical interest on account of their potential for novel structural formulation [18,19], and apoptosis has even been recognised as a side effect of vancomycin in human medicine when used as a therapy to overcome MRSA [20,21].

To assess the natural occurrence of the rat nephropathic potential of European *P*. *polonicum* for usage of that taxonomy across the world, examples of equivalent literature assignments to *P. polonicum* elsewhere have already been questioned [6]. A further example is *Penicillium* fungi from Central and Southern Africa, isolated from Cameroonian maize [22] and South African pig feed [23] and assigned either to *P. crustosum* or *P. polonicum*, although it is not clear whether the latter conformed with the critical *Penicillium* revision in the 1990s [24]. The same partially defined green-fluorescent metabolite [25] was toxic to human epithelial cells, but it bears no apparent resemblance, chemically or pharmacologically, to the present apoptotic *P. polonicum* peptide metabolite(s). Neither of the African fungi seem to be archived anywhere as a single spore isolate to ensure purity. 

The recent study on *P. polonicum* apoptosis [6] was accompanied by some comparison with the well-known nephrotoxin ochratoxin A (OTA), where apoptosis was also recognised by the ApopTag fluorescent staining but OTA seemed to express additional intrinsic toxicity. Recent diagnosis of OTA-induced renal pathology in dogs has been diagnosed as pyroptosis [26], but it is difficult to see how focusing on in vitro responses of dog kidney cells to OTA can correlate to unclear histopathological responses in male mice given OTA via unnatural intraperitoneal injections over 2 weeks. Histology illustration shows no clear evidence of fibrosis, and arrow pointing is unexplained within eosinophilic lumens. Classic mouse renal cancer response to lifetime OTA, exclusively in males, needed much higher doses than for rats [27,28]. Corroboration for female mouse response here could have been helpful, since excretion of small urinary proteins with important social properties in mice and rats and an ability to bind to OTA may contribute to their cortical epithelial salvage and carrier route for toxicity [29]. Authors also have two citations claiming renal accumulation of OTA; the first has nothing to do with whole animals and the second [30], which explains the flaw experimentally, is completely mis-quoted. There seems to be a need for clarification and extent of mechanisms of cell death within mycotoxicology.

## 4. Methods

For animal experimentation, methodology was exactly as previously described [6]. Experimental Sprague–Dawley rats in the Imperial College animal facility (21 °C, 12 h light–dark cycle) were caged on sawdust and given diet pellets (rat and mouse diet 1, Special Diet Services, Essex, UK) and water ad lib. Animals given mycotoxin fraction experimentally in feed were caged individually on absorbent paper, changed daily, and given a powdered diet containing the homogenised experimental material provided in an aluminium dish. The amount of homogenised feed was adjusted to ensure complete daily consumption during a 24 h period, although normally mostly at night, based on animal weight and experience (usually 15–20 g for adults).
